# Bibliographical Notice

**Published:** 1839-07-13

**Authors:** 


### BIBLIOGRAPHICAL NOTICE.

*The New York Quarterly Medical and Surgical Journal.* No. 1. July, 1839.

It has been said that the multiplication of periodicals is adverse to the interests of our medical literature; that the strength which might be concentrated upon a few, is frittered away upon a number of journals, till the talent scattered through each becomes obscured in a general mass of rubbish. Such are the opinions expressed by some of our contemporaries—by the oldest in the field, and by the new-comer of to-day. Our New York friends think that "with the addition of a well-conducted quarterly in that city, there would be as many journals as are needed;" while the elder quarterly would be glad to see the talent of the country rally round it, and unite in sending forth a journal, creditable to the national literature. We differ from these views of our respectable contemporaries. Every addition to the ranks is, we think, a reinforcement of the strength of the whole—not taking from the old, but developing new resources, and not abstracting from the patronage of any, but adding to that of all. Competition is the soul of enterprise in medical literature, as in every other pursuit. The greater the amount of journals from which the reader may choose, the greater will be the exertions of each to secure the choice. Besides, an increased supply will create an increased demand: the great body of our intelligent physicians will find time to read most, if not all, the really good journals. A journal that contains valuable original matter, *must* sustain itself; and there is talent enough in the country, if properly sought out and rewarded, to supply it to twice the existing number.

The first number of the New York Journal contains much excellent matter, of the usual variety—essays, cases, and reviews. Particular criticism would be out of place, but our readers will find in our columns acceptable extracts from the Journal. We have to add the expression of our respect for the Editors, and good wishes for the success of their undertaking.

